# Early Exacerbation Relapse is Increased in Patients with Asthma and Bronchiectasis (a Post hoc Analysis)

**DOI:** 10.1007/s00408-023-00601-1

**Published:** 2023-02-06

**Authors:** Andrew R. Hill, Pallavi Bedi, Manjit K. Cartlidge, Kim Turnbull, Samantha Donaldson, Andrea Clarke, Jane Crowe, Kadiga Campbell, Ruzanna Franguylan, Adriano G. Rossi, Adam T. Hill

**Affiliations:** 1 School of Medicine, University of St. Andrews, North Haugh, St. Andrews, UK; 2 Centre for Inflammation Research, Queen’s Medical Research Institute, 47 Little France Crescent, Edinburgh, UK; 3 Royal Infirmary of Edinburgh, 51 Little France Crescent, Edinburgh, UK

**Keywords:** Asthma, Bronchiectasis, Exacerbations, Intravenous antibiotics

## Abstract

**Purpose:**

Asthma is a common comorbidity in patients with bronchiectasis and has been shown to increase the risk of bronchiectasis exacerbations. This paper explores the impact of comorbid asthma on patients receiving intravenous antibiotic treatment for bronchiectasis exacerbations.

**Methods:**

This was a post hoc analysis of the Meropenem randomised controlled trial of 90 patients that had intravenous antibiotic treatment for bronchiectasis exacerbations. The participants were split into two groups: group 1 (asthma and bronchiectasis) and group 2 (bronchiectasis). The authors assessed response to treatment and time to next exacerbation.

**Results:**

There were 38 participants in group 1 and 34 participants in group 2. The groups were found to be comparable in terms of age, sex, and bronchiectasis severity (median (95% CI) group 1 and then group 2 data): age 64.0(59.3, 68.6) and 63.6(57.9, 69.4) years old, *p* = 0.8; 57.9% and 64.7% female, *p* = 0.6; Bronchiectasis Severity Index 11.1(9.8, 12.4) and 10.1(8.2, 12.0), *p* = 0.3. There was a similar response to treatment between the groups, but group 1 were found to relapse early by day 14, 31.6% in group 1 and 11.8% in group 2, *p* = 0.03. In the Cox proportional hazards model, asthma was the only independent risk factor for early relapse by day 14 (odds ratio (95% CI) 3.16 (1.02–9.79), *p* = 0.047).

**Conclusion:**

The clinical response to treatment was similar but patients with coexisting asthma were at increased risk of early relapse within 14 days of stopping intravenous antibiotic therapy. Clinical Trial Registration: NCT02047773.

## Introduction

Bronchiectasis is a common chronic respiratory condition characterised by regular cough, sputum production and at risk of recurrent respiratory tract infections [1]. It affects around 566 per 100,000 women and 486 per 100,000 men in the UK [2]. The true prevalence of asthma is not known with rates ranging from 1 to 68% [1–4]. The large epidemiology study (18,793 patients) by Quint and colleagues quoted 42% had asthma in their bronchiectasis cohort [2].

In a retrospective study with 463 patients, asthma was an independent risk factor for increased bronchiectasis exacerbations [5]. This was confirmed in a small prospective study [6]. In a systematic review exploring the impact of bronchiectasis on co-existent asthma, the mean prevalence of bronchiectasis was 37% of 839 patients with asthma. Asthma patients with comorbid bronchiectasis were shown to have more frequent exacerbations [7].

A European consensus agreement indicated that antibiotics are recommended for exacerbations in patients with bronchiectasis with a deterioration in three or more of the following key symptoms for at least 48 h: cough, sputum volume and/or consistency, sputum purulence, breathlessness and/or exercise tolerance, fatigue and/or malaise, and haemoptysis [8]. Intravenous antibiotics are indicated for an infective exacerbation of bronchiectasis when patients are particularly unwell, have resistant organisms, or have failed to respond to oral therapy [9].

The aim of this study was to explore whether co-existent asthma influenced the response to intravenous antibiotics. End points of interest were response rates to antibiotic therapy and impact on the time to next needing further antibiotic treatment.

## Study Design and Methods

This was a post hoc analysis of the meropenem study exploring the impact of shortening antibiotic therapy for exacerbations of bronchiectasis requiring intravenous antibiotic therapy [10]. The impact of co-existent asthma was not studied in the original publication. End points of interest were response to antibiotic therapy and time to next exacerbation requiring antibiotic therapy.

The Meropenem study was a randomised control trial consisting of 90 participants that all received intravenous antibiotic treatment for bronchiectasis exacerbations. This post hoc study analysed the data from the meropenem study looking at the response to treatment comparing people with bronchiectasis with and without comorbid asthma. The participants were split into two groups: group 1 (asthma and bronchiectasis) and group 2 (bronchiectasis).

Asthma was diagnosed in this study based on respiratory clinic diagnosis and these patients had both asthma and bronchiectasis coded on their diagnostic list. Patients with COPD were excluded. In this post hoc study the authors did not explore the asthma diagnosis further.

Meropenem (2 g TDS) was chosen for the intravenous antibiotic treatment as it is broad spectrum and was suitable for all participants. All participants were able to complete the full treatment and did not receive any other treatment for their bronchiectasis exacerbations, in particular no patient received concomitant systemic corticosteroids.

The authors assessed response to treatment at day 14 compared with baseline before antibiotic therapy by sputum purulence (purulent to mucopurulent, mucoid or no sputum; or mucopurulent to mucoid or no sputum) [11], blood tests (full blood count, C-Reactive Protein, erythrocyte sedimentation rate), bacterial clearance and bacterial load [12], lung function (forced expired volume in 1 s and forced vital capacity), incremental shuttle walk test, quality of life questionnaires (St George’s Respiratory Questionnaire (4 unit or greater decrease) [13], Leicester Cough Questionnaire (1.3 unit or greater increase) [14], and time to next exacerbation requiring further antibiotic therapy.

### Statistical Analysis

For demographic and clinical variables, the authors presented data as median (interquartile range IQR) for continuous variables and number (%) for categorical variables, unless otherwise stated. For the secondary end points, to compare the proportion of participants with clinical improvement a binomial test for the comparison of proportions has been used. Time to next exacerbation is shown using a Kaplan–Meier survival curve with group comparisons using a log-rank statistic. A multivariable Cox proportional hazards model was generated for time to next exacerbation with the following variables: treatment (14 days versus shorter treatment), Bronchiectasis Severity Index (which includes data on age, Body Mass Index, FEV_1_% predicted, hospital admission in the last 2 years, number of exacerbations in the previous year, MRC Breathlessness Score, Pseudomonas colonisation, colonisation with other organisms and radiological severity) (BSI mild 0–4, moderate 5–8 and severe 9 or more) and coexisting asthma (yes, no). Data were analysed using SPSS version 25; significance was accepted with p values: *p < 0.05.

## Results

From the original 90 patients, 72 were included in this study. We excluded 12 patients with COPD and 6 patients with coexisting COPD and asthma. The baseline data for patients with bronchiectasis with asthma (group 1) and without coexisting asthma (group 2) are shown in Table 1.

**Table 1:** Baseline demographics of study participants

	Asthma and bronchiectasis (Group 1)	Bronchiectasis without asthma (Group 2)	*p* value
Numbers	38	34	
Age (years)	64.0 (59.3, 68.6)	63.6 (57.9, 69.4)	0.76
Sex			
Female	22 (57.9%)	22 (64.7%)	0.55
Male	16 (42.1%)	12 (35.3%)	
Smoking status			
Never	27 (71.1%)	28 (82.4%)	0.28
Ex-smokers (greater or more than 1 year)	11 (28.9%)	6 (17.6%)	
Current Smokers or smokers less than 1 year	0	0	
Place of intravenous antibiotics			
Domiciliary	30 (79.0%)	27 (79.4%)	
In hospital	8 (21.0%)	7 (20.6%)	1
Treatment length			
14 days	22 (57.9%)	15 (44.1%)	0.24
Shortened treatment	16 (42.1%)	19 (55.9%)	
Duration of shortened treatment (days)	7 (7, 8.50)	7 (7, 7)	0.51
Aetiology			
Idiopathic	21 (55%)	15 (44%)	0.34
Post infectious	8 (21%)	11 (32%)	0.3
ABPA	3 (8%)	0 (0%)	0.24
Immune defect	2 (5%)	3 (9%)	0.66
Inflammatory arthritis	1 (3%)	0 (0%)	1
Ciliary defect	2 (5%)	1 (3%)	1
Inflammatory bowel disease	0 (0%)	1 (3%)	0.47
Interstitial lung disease	0 (0%)	3 (9%)	0.1
Other	1 (3%)	0 (0%)	1
WCC (× 10^9^/L)	7.50 (6.28, 8.72)	7.95 (5.95, 10.1)	0.73
Neutrophils (× 10^9^/L)	4.64 (3.90, 5.81)	4.66 (3.77, 6.88)	0.78
Lymphocytes (× 10^9^/L)	1.80 (1.43, 2.20)	1.72 (1.22, 1.99)	0.21
Monocytes (× 10^9^/L)	0.60 (0.53, 0.77)	0.70 (0.53, 0.89)	0.19
Eosinophils (× 10^9^/L)	0.20 (0.10, 0.30)	0.14 (0.088, 0.21)	0.1
CRP (mg/L)	8.00 (4.00, 18.0)	9.00 (3.00, 19.0)	0.98
ESR (mm/hr)	13.0 (7.00, 32.8)	21.0 (8.00, 34.0)	0.39
Colonized with			
*Pseudomonas aeruginosa*	19 (50%)	18 (53%)	0.01
Other pathogens	19 (50%)	10 (29%)	
No growth/mixed normal flora	0 (0%)	6 (18%)	
Bacterial load (cfu/Ll)	7.1 × 10^7^ (9.8 × 10^6^, 2.5 × 10^8^)	1.4 × 10^7^ (5.0 × 10^6^, 2.0 × 10^8^)	0.08
On inhaled steroids	36 (94.7%)	17 (50.0%)	< 0.0001
Inhaled steroid dose (beclometasone equivalent in mcg)	2000 (1000, 2000)	2000 (1600, 2000)	0.22
On long-term oral steroids	2 (5.3%)	1 (2.9%)	1
On long-term antibiotics			
Oral	4 (10.5%)	0 (0%)	0.12
Inhaled	1 (2.6%)	4 (11.8%)	0.18
Intravenous	1 (2.6%)	2 (5.9%)	0.6
ISWT (m)	260 (130, 450)	305 (165, 422)	0.86
FEV_1_ (L)	1.64 (1.20, 2.50)	1.51 (1.09, 1.84)	0.24
FEV_1_ (% predicted)	70.0 (53.0, 79.0)	66.5 (54.5, 83.5)	0.6
FVC (L)	2.46 (2.08, 3.40)	2.25 (1.87, 2.88)	0.21
FVC (% predicted)	82.0 (65.0, 98.5)	78.0 (70.0, 95.5)	0.98
Radiological severity			
3 or more lobes affected or cystic bronchiectasis in any lobe	24 (63.2%)	24 (70.6%)	0.62
BSI			
Total score	11.1(9.79, 12.4)	10.1 (8.22, 12.0)	
Mild	0 (0%)	6 (17.6%)	0.27
Moderate	13 (34.2%)	9 (26.5%)	
Severe	25 (65.8%)	19 (55.9%)	
LCQ (units)	10.2 (7.82, 13.0)	11.8 (8.80, 15.4)	0.14
SGRQ (units)	48.4 (30.8, 64.4)	36.1 (21.2, 49.7)	0.06

Data presented as median (interquartile range) or number (percentage)

*ABPA* allergic bronchopulmonary aspergillosis; *WCC* white cell count; *CRP* c-reactive protein; *ESR* erythrocyte sedimentation rate; *ISWT* Incremental shuttle walk test; *FEV*_1_ Forced expiratory volume in 1 s; *FVC* Forced vital capacity; *BSI* Bronchiectasis Severity Index; *LCQ* Leicester Cough Questionnaire; *SGRQ* St. George’s Respiratory Questionnaire

There was no significant difference between the groups at baseline with age, sex, smoking status, place that they received intravenous antibiotics, duration of antibiotic therapy, aetiology, full blood count and inflammatory markers (erythrocyte sedimentation rate and c-reactive protein), quantitative bacterial load, inhaled steroid dose, oral steroids use, long-term antibiotic use, spirometry, incremental shuttle walk test, radiological severity, Bronchiectasis Severity Index, and quality of life questionnaires. Group 1 patients were however, all colonised with pathogens whereas only 82% of patients in group 2 were colonised (*p* = 0.01). Patients with coexisting asthma had a higher proportion that were on inhaled corticosteroids (94.7% vs 50.0%, *p* < 0.0001). (Table 1).

When assessing response to treatment from baseline to 14 days after starting treatment there was similar improvement in sputum colour, full blood count, erythrocyte sedimentation rate, c-reactive protein, bacteriology including quantitative bacterial load, spirometry, incremental shuttle walk test, and quality of life between the groups (Table 2).

**Table 2:** Difference from baseline to 14 days after starting treatment and clinical recovery

	Group 1	Group 2	*p* value
Numbers	38	34	
FEV_1_ (L)	0.020 (− 0.14, 0.14)	0.035 (− 0.038, 0.20)	0.35
FEV_1_ (% predicted)	3.0 (− 2.5, 9.0)	1.0 (− 1.0, 7.5)	0.87
FVC (L)	0.040 (− 0.13, 0.34)	0.14 (− 0.060, 0.38)	0.53
FVC (% predicted)	3.5 (− 1.0, 14.8)	4.5 (− 2.8, 10.5)	0.78
ISWT (m)	0.0 (− 20.0, 32.5)	30.0 (− 10.0, 90.0)	0.12
ESR (mm/h)	− 1.0 (− 9.0, 3.0)	− 1.0 (− 15.5, 4.0)	1
Neutrophils (× 10^9^/L)	− 0.98 (− 2.01, 0.32)	− 0.74(− 2.82, 0.072)	0.68
Lymphocytes (× 10^9^/L)	− 0.085 (− 0.42, 0.14)	− 0.14 (− 0.40, 0.062)	0.59
Monocytes (× 10^9^/L)	− 0.025 (− 0.13, 0.14)	− 0.10 (− 0.23, 0.045)	0.03
Eosinophils (× 10^9^/L)	0.05 (− 0.03, 0.14)	0.06 (− 0.01, 0.18)	0.86
CRP (mg/L)	− 3.0 (− 16.0, 1.0)	− 1.0 (− 7.0, 5.2)	0.34
ESR (mm/h)	− 1.0 (− 9.0, 3.0)	− 1.0 (− 15.5, 4.0)	1
Colonized with			
*Pseudomonas aeruginosa*	7 (18%)	6 (18%)	0.13
Other pathogens	18 (47%)	10 (29%)	
No growth/mixed normal flora	11 (29%)	18 (53%)	
Missing data	2 (5%)	0 (0%)	
Log10 bacterial load reduction (cfu/ml)	2.25 (0.01, 6.66)	3.70 (− 0.04, 7.83)	0.53
Sputum colour			
Improved	47.20%	39.30%	
Stable	41.70%	50%	Stable
Deteriorate	11.10%	10.70%	
Stable or improved	88.90%	89.30%	
LCQ			
Clinical improvement	48.60%	63.30%	0.23
No clinical improvement	51.40%	36.70%	
SGRQ			
Clinical improvement	51.40%	53.10%	0.89
No clinical improvement	48.60%	46.90%	
LCQ or SGRQ			
Clinical improvement	69.40%	84.40%	0.17
No clinical improvement	30.60%	15.60%	

Data presented as median (interquartile range) or percentage. Clinical recovery for the questionnaires were defined as a 1.3 unit or greater increase in LCQ or 4 unit or greater decrease in SGRQ

*FEV*_1_ Forced expiratory volume in 1 s; *FVC* Forced vital capacity; *ISWT* Incremental shuttle walk test; *WCC* white cell count; CRP c-reactive protein; *ESR* erythrocyte sedimentation rate; *LCQ* Leicester Cough Questionnaire; *SGRQ* St. George’s Respiratory Questionnaire

**Fig. 1:**
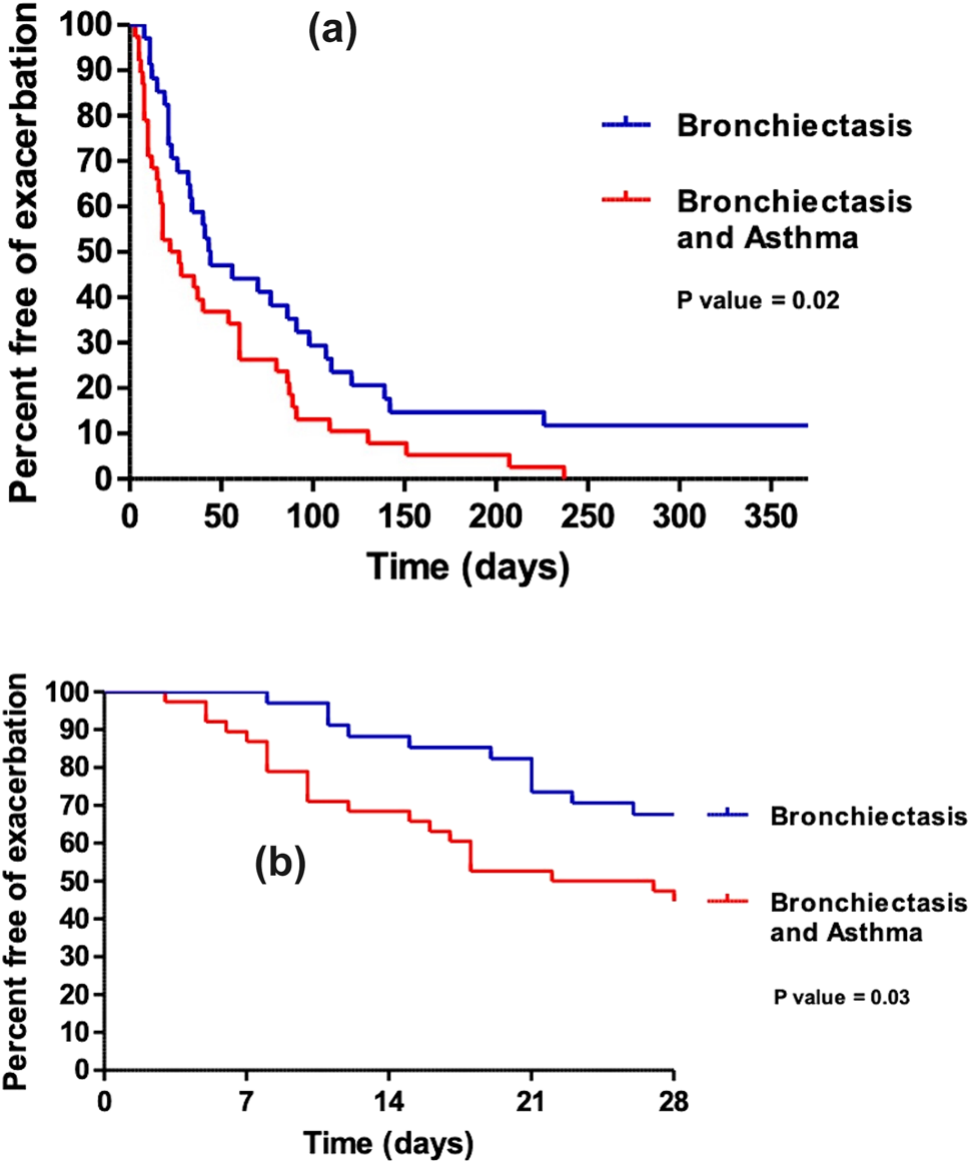
**a** Kaplan Meier plot to estimate the time to next exacerbation up to 365 days after completing treatment comparing bronchiectasis with and without asthma. *p* = 0.02. **b** Kaplan Meier plot to estimate the time to next exacerbation up to 28 days after completing treatment comparing bronchiectasis with and without asthma. *p* = 0.03

Patients with comorbid asthma with bronchiectasis had a shortened time to next exacerbation over 365 days, log-rank *p* = 0.02 (Fig. 1a). It appeared from analyses from the curves the biggest change between the groups was in the first 28 days (see Fig. 1b). The authors proceeded with a Cox proportional hazards model adjusting for confounders identified in the original trial (Bronchiectasis Severity Index and duration of treatment) but in addition coexisting asthma was added. Over a 365 days period, the independent risk factors for time to next exacerbation were longer duration of antibiotics having a shortened time to next exacerbation (hazard ratio (95% CI) 1.94 (1.19–3.16), *p* = 0.008)) and mild bronchiectasis having a prolonged time to next exacerbation (hazard ratio (95% CI) 0.34 (0.12–0.95), *p* = 0.039)). Coexisting asthma was not an independent risk factor over a 365-day period and there were no independent factors identified over a 28-day period after completing treatment. Asthmatics were, however, at a higher risk of exacerbation within 14 days after completing treatment (Hazard Ratio (HR) (95% CI) 3.16 (1.02–9.79), *p* = 0.047 compared to those with bronchiectasis alone. Duration of treatment and bronchiectasis severity were not independent risk factors by day 14 after finishing antibiotics (see Table 3).

**Table 3:** Cox proportional hazards model

	Independent risk factors for an exacerbation	Odds ratio	*p* value
Day 14	Asthma	3.16 (95% 1.02–9.79)	0.047
Day 365	Mild bronchiectasis	0.34 (95% 0.12–0.95)	0.039
	14-days treatment	1.94 (95% 1.19–3.16)	0.008

## Discussion

In this study, we found asthma was a common comorbidity in exacerbations of bronchiectasis that required intravenous antibiotics. There was also a small cohort that had COPD and bronchiectasis, but these were excluded from the post hoc analysis. There was a similar clinical response to intravenous antibiotic in terms of recovery at 14 days. Patients with coexisting asthma however relapsed quicker. On multivariable modelling asthma was an independent risk factor for early relapse. This study showed that relapse was early within 14 days of stopping antibiotic treatment.

Asthma is a common comorbidity of bronchiectasis and has been shown to increase the risk of bronchiectasis exacerbations [1–6]. Understanding how asthma may impact treatment/s for bronchiectasis is important. Diagnosing asthma using lung function tests in patients with bronchiectasis, however, can be challenging as patients with bronchiectasis can have normal lung function, obstructive lung disease or restrictive lung disease [15]. In this study the asthma diagnosis was confirmed in a secondary care clinic and around 95% were on long-term inhaled steroids. There however, wasn’t a significant difference in the eosinophil count between the groups. In this post hoc study the authors did not explore the asthma diagnosis further.

In this study patients were recruited with a bronchiectasis infective exacerbation as the predominant diagnosis. Thus, all the patients were selected as they were thought to be having an exacerbation of their bronchiectasis rather than an exacerbation of asthma. This was supported with no patients requiring oral steroid at the start of the exacerbation. In addition, all groups responded well to intravenous antibiotic treatment and none of the asthma patients required oral steroids during therapy up to 14 days.

When comparing both groups, the baseline characteristics were similar (age, sex, smoking status, place of intravenous antibiotics therapy, duration of intravenous antibiotics, aetiology, white cell count and differential white cell count, c-reactive protein, erythrocyte sedimentation rate, bacterial load, inhaled steroid dose, number on long-term oral steroids, number on long-term antibiotics, incremental shuttle walk test, spirometry, radiological severity, baseline Bronchiectasis Severity Index, Leicester Cough Questionnaire and St. George’s Respiratory Questionnaire). The asthma group however had a higher percentage on inhaled steroids (95% vs 50%). Despite national guidelines, it is well recognised that many patients with bronchiectasis without coexisting asthma and COPD are treated with inhaled steroids [1, 16]. The asthma group at baseline had a higher percentage colonisation but there was a similar bacterial load between the groups, the differences are unlikely to be clinically significant.

When assessing response to treatment the two groups responded very similarly to the intravenous antibiotic treatment in all parameters studied. There was no significant difference between the spirometry, full blood count, c-reactive protein, erythrocyte sedimentation rate, bacteriology, bacterial load, incremental shuttle walk test, and quality of life questionnaires.

In the initial Kaplan Meier curve patients with bronchiectasis and asthma relapsed quicker. On exploring the graph an earlier relapse was driving the difference between the curves. In the Cox proportional hazard models, the independent risk factors for an early exacerbation were a longer course of intravenous antibiotic therapy and having a higher severity of bronchiectasis as in the original paper [10]. There was, however, no independent risk factors identified for relapse at day 28 after completing treatment. At day 14 after completing treatment, asthma was the only independent risk factor for early relapse. A hypothesis for these findings could be that although the initial bronchiectasis exacerbation responded well to antibiotic therapy, there may have been a secondary asthma exacerbation. This study, however, only recorded when the patient next received antibiotic therapy, and we therefore don’t have any further details. Further longitudinal studies would be needed to explore this hypothesis.

A hypothesis is that early relapse in patients with bronchiectasis and asthma was because they did not receive systemic steroids in addition to the intravenous antibiotics. A possibility could be that patients with coexisting asthma that are having a bronchiectasis exacerbation could be treated with systemic steroids along with the intravenous antibiotics. Randomised control trials are needed to explore this further.

### Limitations

This was a post hoc analysis of the original study and therefore has not been powered to detect the difference between the two groups. The asthma diagnosis was not formally explored by the authors, but the asthma diagnosis was confirmed in a secondary bronchiectasis clinic. Another limitation was the relapse after the initial bronchiectasis therapy was taken from participant and general practice follow up. There was, however, no study clinician assessment of the relapse after the initial treatment and therefore, we do not have further details about that relapse.

## Conclusion

In this post hoc analysis study asthma was a common comorbidity of bronchiectasis exacerbations that needed intravenous antibiotic therapy. Both groups had a similar clinical response to intravenous antibiotics for bronchiectasis exacerbations. Asthmatics had however, an increased risk of early relapse within 14 days of completing treatment. Randomised control trials are needed to explore whether patients with bronchiectasis and coexisting asthma would benefit with adjunctive systemic steroids with intravenous antibiotics.

## Abbreviations

ABPA: Allergic bronchopulmonary aspergillosis
WCC: White cell count
CRP: C-Reactive protein
ESR: Erythrocyte sedimentation rate
ISWT: Incremental shuttle walk test
FEV_1_: Forced expiratory volume in 1 s
FVC: Forced vital capacity
BSI: Bronchiectasis Severity Index
LCQ: Leicester Cough Questionnaire
SGRQ: St George’s Respiratory Questionnaire
COPD: Chronic Obstructive Pulmonary Disease
